# Supplementary material for: Uncovering miRNA-mRNA Regulatory Modules in Developing Xylem of Pinus massoniana via Small RNA and Degradome Sequencing
Source: Int J Mol Sci. 2021 Sep 21;22(18):10154. doi: 10.3390/ijms221810154 (PMC8472836; doi:10.3390/ijms221810154)
Supplement: Supplementary file 1 [file ijms-22-10154-s001.zip › ijms-1365557-supplementary/Additional file S2.zip!/Additional file 2/novel_88_Cluster-18410.90022_224_TPlot.pdf]

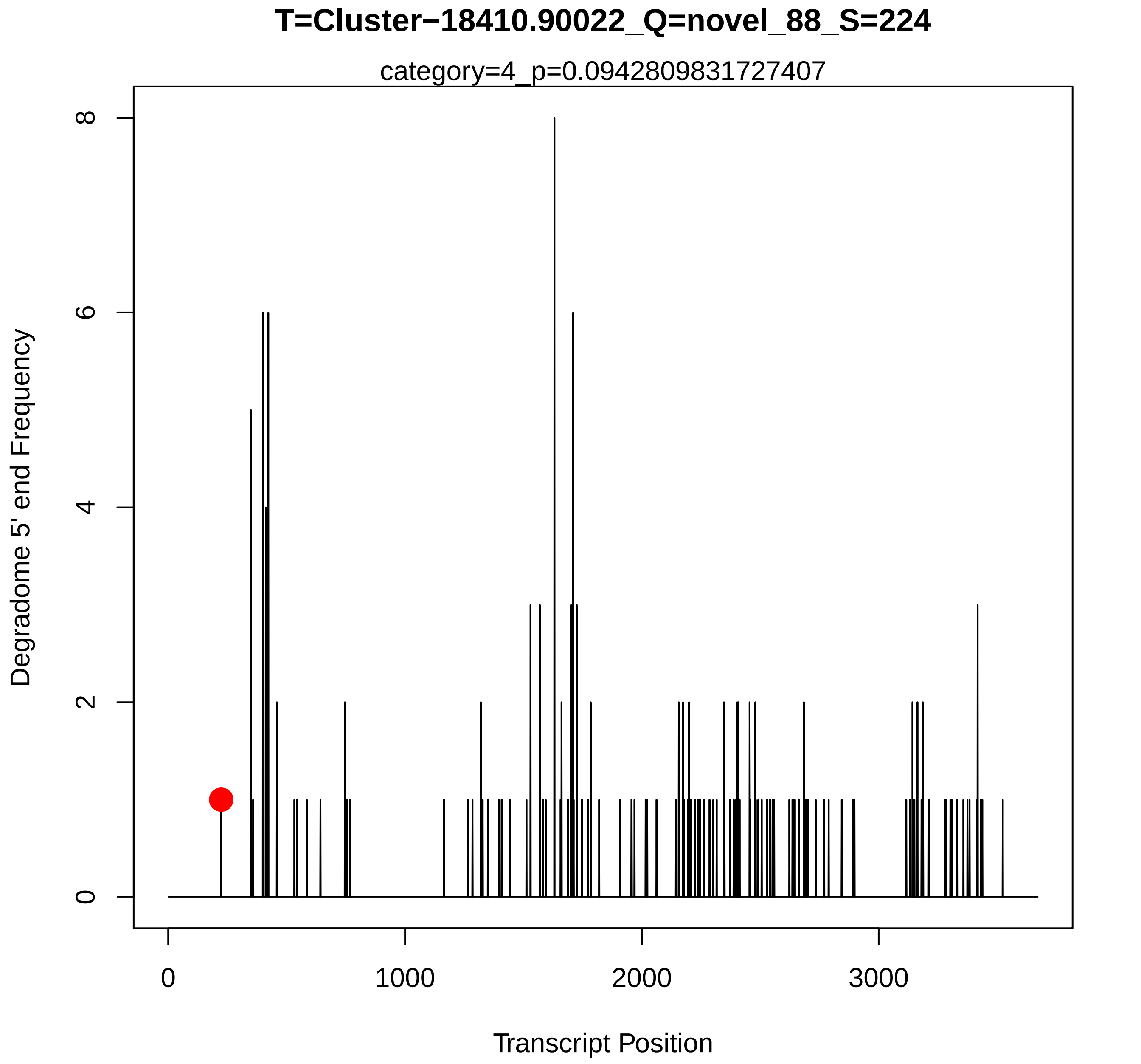

T=Cluster−18410.90022_Q=novel_88_S=224
category=4_p=0.0942809831727407
Degradome 5' end Frequency
Transcript Position
0
2
4
6
8
0
1000
2000
3000
